# Association between HOTAIR Polymorphisms and Lymphoma

**DOI:** 10.31557/APJCP.2021.22.9.2831

**Published:** 2021-09

**Authors:** Sara Kashani, Hosein Ali Sasan, Gholamreza Bahari, Behrouz Mollashahi, Seyed Mahdi Hashemi, Mohsen Taheri

**Affiliations:** 1 *Department of Biology, Faculty of Science, Shahid Bahonar University, Kerman, Iran. *; 2 *Children and Adolescent Health Research Center, Resistant Tuberculosis Institute, Zahedan University of Medical Sciences, Zahedan, Iran. *; 3 *Genetics of Non-communicable Disease Research Center, Zahedan University of Medical Sciences, Zahedan, Iran.*; 4 *Department of Internal Medicine, School of Medicine, Zahedan University of Medical Sciences, Zahedan, Iran. *; 5 *Department of Genetic, School of Medicine, Zahedan University of Medical Sciences, Zahedan, Iran. *

**Keywords:** Long non, coding RNA, HOTAIR, polymorphism, Lymphoma

## Abstract

**Introduction:**

The long non-coding RNA, HOTAIR, involved in cancer initiation and development.

**Objective:**

The aim of present study was to investigate the association of single nucleotide polymorphisms in the HOTAIR gene with lymphoma.

**Methods:**

We conducted a case-control study of 156 individuals with non-Hodgkin Lymphoma (NHL), 53 individuals with Hodgkin’s lymphoma (HL), and 245 unrelated healthy individuals to identify the genotype frequencies of each polymorphism. Genotyping of the SNPs (rs920778 T>C, rs1899663 G>T, rs4759314 A>G and rs12826786 C>T) was carried out using the polymerase chain reaction-restriction fragment length polymorphism.

**Results and Conclusion:**

The finding showed that rs1899663 variant of HOTAIR gene significantly decreased the risk of NHL in codominant, dominant, over-dominant and allelic inheritance models. We did not find any association between HOTAIR rs12826786, rs920788 and rs4759314 variants and NHL. The results indicated that neither the overall chi-square comparison of the cases and controls, nor the logistic regression analysis showed any association between HOTAIR polymorphisms and HL. Conclusively, our findings showed that rs1899663 of HOTAIR significantly decreased the risk of non-Hodgkin Lymphoma.

## Introduction

Cancer is a major cause of morbidity and mortality throughout the world. Lymphoma is the most common malignant neoplasms of the lymphatic system originating in the lymphoid hematopoietic system, representing 3.7% of all cancer diagnoses in Iran (https://gco.iarc.fr/today/data/factsheets/populations/364-iran-islamic-republic-of-fact-sheets.pdf , Shilkofski et al., 2020). Lymphoma can be divided into two groups Hodgkin’s lymphoma (HL) and non-Hodgkin’s lymphoma (NHL) (Lin et al., 2018). According to the GLOBOCAN database, NHL with 509,590 new cases and 248,724 deaths in 2018 is one of the common cancers in worldwide (Bray et al., 2018). However, the exact pathogenesis of NHL is still not fully understood but many risk factors reported that are involved in lymphoma development. Environments as well as genetic factors are two major risk factors for lymphoma (Zhong et al., 2019; Tang et al., 2020). 

Long-chain noncoding RNA (lncRNA) is a class of RNA with more than 200 nucleotides in length which does not encode any proteins (regulatory non-coding RNAs) (Bayram et al., 2015;Minn et al., 2020). They play a key role in a range of biological processes including transcriptional regulation, posttranscriptional regulation, translation regulation, and chromatin remodeling (Ren et al., 2020). lncRNAs is now known to be causally involved in cancers as either oncogenes or tumor suppressor genes (Su et al., 2018). 

HOTAIR (HOX transcript antisense intergenic RNA) is one of the most well-known lncRNAs with a length of 2158 bp which located on chromosom 12 (Moazeni-Roodi et al., 2020). HOTAIR interact with Polycomb Repressive Complex 2 (PRC2) acting as a molecular scaffold that modify chromatin remodeling (Yuan et al., 2020). It seems that chromatin remodeling through PRC2 cause cancer development such as hematologic malignancies (Oh et al., 2016). Many studies have demonstrated that HOTAIR expression was increased in several different types of cancers including breast cancer (Zhao et al., 2018), lung cancer (Liu et al., 2018) and acute leukemia (Hao and Shao, 2015). In addition it has been reported that over-expression of HOTAIR correlate with diagnosis and prognosis of acute leukemia and lymphoma (Lin et al., 2018). It has been showed that single nucleotide polymorphisms alter the expression and function of lncRNAs (Rajagopal et al., 2020). Several epidemiological investigations demonstrate an association between HOTAIR polymorphisms and cancers however, the findings were inconsistent and controversial (Qiu et al., 2016; Hassanzarei et al., 2017; Jiang et al., 2019; Moschovis et al., 2019; Li et al., 2020). In the present study we aimed to determine the possible association between HOTAIR polymorphisms and lymphoma in a sample of Iranian population. 

## Materials and Methods

From June 2018 through May 2020, all patients with lymphoma that diagnosed and treated at the Department of internal medicine of the Ali ibn Abi Talib hospital (regional referral hospital for cancer cases in the southeast of Iran) were considered. 156 patients with NHL, 53 patients with HL and 245 healthy individuals enrolled in the study. Diagnosis was based on clinical sign, histopathological and immunohistochemical findings according to the World Health Organization classification. The control group had no cancer history. They were genetically unrelated to the cases, and were selected from the same ethnicity as patients. All participants were all from Sistan & Baluchistan (Southeast of Iran). The project was approved by the ethics committee of the Zahedan University of Medical Sciences (IR.ZAUMS.REC.1400.037) and informed consent was taken from all contributors. For DNA isolation 2 ml of venous blood were taken from all participants and DNA extraction was performed by salting out method. The concentration and quality of the extracted DNA was assessed using NanoDrop2000 (Thermo Fisher Scientific, Waltham, MA, USA). Genotyping of HOTAIR variants (rs920778 T>C, rs1899663 G>T, rs4759314 A>G and rs12826786 C>T) was carried out using the polymerase chain reaction-restriction fragment length polymorphism (PCR-RFLP). Primer sequences, restriction enzymes and length of the fragments are summarized in Table 1. PCR was done in a 0.20 ml PCR reaction tube containing 1 μl of genomic DNA (~100 ng/ml), 1 μl of each primer, 8 μl of mastermix and 6 μl ddH2O. PCR cycling condition was as follow: initial denaturation at 95°C for 5 min followed by 30 cycles of 30 s at 95°C, optimal annealing temperature (Table 1) for 30 s and 30 s at 72°C, with a final extension of 72°C for 10 min. The PCR product was digested by suitable restriction enzyme (Table 1) and analyzed by gel electrophoresis on 2.5% agarose gel. For quality control, we randomly selected 10% of the samples to repeat the results. 

*Statistical Analysis*

Statistical calculations were made with the use of SPSS 20 software (SPSS Inc., Chicago, IL, USA). We evaluated the differences between cases and controls in demographic characteristics and genotype results by chi-square test. The associations between IL1 variants and lymphoma risk were evaluated by odds ratios (ORs) and 95% confidence intervals (CIs) under different genetic models. Differences were considered to be statistically significant when P<0.05.

## Results

This case control study included 156 patients with NHL (96 males and 60 females; mean age, 45.13±15.99 years), 53 patients with HL (30 males and 23 females; mean age, 36.66±15.51 years), and 245 healthy subjects (142 males and 103 females; mean age, 48.5 ± 11.4). The genotype and allelic frequencies of HOTAIR variants in NHL patients and controls are presented in Table 2. The finding revealed that rs1899663 variant of HOTAIR gene significantly decreased the risk of NHL in codominant (OR = 0.22, 95% CI = 0.13–0.36, p < 0.001, GT vs GG), dominant (OR = 0.25, 95% CI = 0.15–0.41, p < 0.0001, GT+TT vs GG), overdominant (OR = 0.30, 95% CI = 0.19– 0.46, p < 0.0001, GT vs GG+TT) and allelic (OR = 0.66, 95% CI = 0.49–0.88, p =0.005, T vs G) inheritance models. The results demonstrated that HOTAIR rs12826786, rs920788 and rs4759314 variants were not associated with risk of/protection from NHL in codominant, dominant and recessive tested inheritance models (Table 2). Table 3 demonstrates the genotype and allele frequencies of HOTAIR polymorphisms in HL patients and healthy subjects. The findings revealed that neither the overall chi-square comparison of the cases and controls, nor the logistic regression analysis showed any association between HOTAIR polymorphisms and HL. HOTAIR polymorphisms were not associated with the risk of HL in codominant, dominant and recessive tested inheritance models. 

**Table 1:** The Primers Used for Detection of *HOTAIR* Polymorphisms Using PCR-RFLP Methods

HOTAIR polymorphism	Primer sequence (5`->3`)	Restriction Enzyme	Annealing Temperature	Fragment (bp)
Rs920778 T>C				
Forward	F: TTACAGCTTAAATGTCTGAATGTTCC	MspI	60	T allele, 140;
Reverse	R: GCCTCTGGATCTGAGAAAGAAA			C allele, 113+27
Rs1899663 G>T				
Forward	F: TTTTCCAGTTGAGGAGGGTGGA	HphI	66	T allele, 114;
Reverse	R: CTAATGGCAAGGGAAGGGAAGG			G allele, 79+35
Rs4759314 A>G				
Forward	F: TTCAGGTTTTATTAACTTGCATCAGC	AluI	58	G allele, 124;
Reverse	R: ACCCAAAACCATTTCCTGAGAG			A allele, 98+25
Rs12826786 C>T				
Forward	F: GTCCAGTCGCTCGTCCCTGAG	MboI	68	C allele, 337
Reverse	R: AAATCACCTGCTGGCAACGG			T allele, 225+112

**Table 2:** The Genotypes and Allele Distribution of Hotair Polymorphisms in in NHL and Healthy Subjects (Control)

	Case			Control					
rs1899663 polymorphism									
Codominant									
GG	61 (39.1)			34 (13.9)		1			
GT	77 (49.4)			195 (79.6)		0.22 (0.13-0.36)		<0.001	
TT	18 (11.5)			16 (6.5)		0.63 (0.28-1.39)		0.305	
Dominant									
GG	61 (39.1)			34 (13.9)		1			
GT+TT	95 (60.9)			211 (86.1)		0.25 (0.15- 0.41)		<0.001	
Recessive									
GG+GT	138 (88.5)			229 (93.5)		1			
TT	18 (11.5)			16 (6.5)		1.87 (0.92- 3.78)		0.097	
Over dominant									
GG+TT	79 (50.6)			50 (20.4)		1			
GT	77 (49.4)			195 (79.6)		0.30 (0.19- 0.46)		<0.001	
Alleles									
G	199 (63.8)			263 (53.7)		1			
T	113 (36.2)			227 (46.3)		0.66 (0.49- 0.88)		0.005	
rs12826786									
Codominant									
CC	48 (30.8)			47 (22.9)		1			
CT	90 (57.7)			127 (62.0)		0.69 (0.42- 1.12)		0.172	
TT	18 (11.5)			31 (15.1)		0.56 (0.28- 1.15)		0.157	
Dominant									
CC	48 (30.8)			47 (22.9)		1			
CT+TT	108 (69.2)			158 (77.1)		0.66 (0.41- 1.07)		0.116	
Recessive									
CC+CT	138 (88.5)			174 (84.9)		1			
TT	18 (11.5)			31 (15.1)		0.73 (0.39- 1.36)		0.355	
Over dominant									
CC+TT	66 (42.3)			78 (38.0)		1			
CT	90 (57.7)			127 (62.0)		0.83 (0.54- 1.28)		0.448	
Alleles									
C	186 (59.6)			221 (53.9)		1			
T	126 (40.4)			189 (46.1)		0.79 (0.58- 1.06)		0.13	
rs920788 Polymorphism									
Codominant									
TT	98 (62.8)			156 (60.2)		1			
CT	49 (31.4)			96 (37.1)		0.81 (0.53-1.24)		0.397	
CC	9 (5.8)			7 (2.7)		2.04 (0.73-5.67)		0.255	
Dominant									
TT	98 (62.8)			156 (60.2)		1			
CT+CC	58 (37.2)			103 (39.8)		0.89 (0.59- 1.34)		0.677	
Recessive									
TT+CT	147 (94.2)			252 (97.3)		1			
CC	9 (5.8)			7 (2.7)		1.32 (0.48-3.66)		0.617	
Over dominant									
TT+CC	107 (68.6)			163 (97.3)		1			
CT	49 (31.4)			96 (37.1)		0.77 (0.51- 1.18)		0.287	
Alleles									
T	245 (78.5)			408 (78.8)		1			
C	67 (21.5)			110 (21.2)		1.01 (0.72- 1.42)		0.93	
	Case			Control					
rs4759314polymorphism								
Codominant								
AA		151 (96.8)	247 (98.4)		1			
AG		5 (3.2)	4 (1.6)		2.04 (0.54- 7.73)		0.313	
GG		0 (0.0)	0 (0.0)		------		-----	
Alleles								
A		307 (98.4)	498 (99.2)		1			
G		5 (1.6)	4 (0.8)		2.02 (0.54- 7.61)		0.315	

**Table 3:** Genotype and Allele Frequencies of the IL1RN VNTR Polymorphism in HL and Healthy Subjects (Control)

	Case	Control			
rs1899663 polymorphism					
Codominant					
GG	8 ( 15.1)	34 ( 13.9)	1		
GT	37 ( 69.8)	195 ( 79.6)	0.80 ( 0.34-1.87)		0.65
TT	8 ( 15.1)	16 ( 6.5)	2.12 ( 0.67-6.68)		0.238
Dominant					
GG	8 ( 15.1)	34 ( 13.9)	1		
GT+TT	45 ( 84.9)	211 ( 86.1)	0.90 ( 0.39- 2.08)		0.828
Recessive					
GG+GT	45 ( 84.9)	229 ( 93.5)	1		
TT	8 ( 15.1)	16 ( 6.5)	2.54 ( 1.02- 6.30)		0.05
Over dominant					
GG+TT	16 ( 30.2)	50 ( 20.4)	1		
GT	37 ( 69.8)	195 ( 79.6)	0.71 ( 0.37- 1.36)		0.299
Alleles					
G	53 ( 50.0)	263 ( 53.7)	1		
T	53 ( 50.0)	227 ( 46.3)	0.86 ( 0.56- 1.31)		0.52
rs12826786					
Codominant					
CC	14 ( 26.5)	47 ( 22.9)	1		
CT	33 ( 62.2)	127 ( 62.0)	0.87 ( 0.42- 1.77)		0.715
TT	6 ( 11.3)	31 ( 15.1)	0.64 ( 0.22- 1.87)		0.586
Dominant					
CC	14 ( 26.5)	47 ( 22.9)	1		
CT+TT	39 ( 73.5)	158 ( 77.1)	0.82 ( 0.41- 1.65)		0.59
Recessive					
CC+CT	47 ( 88.7)	174 ( 84.9)	1		
TT	6 ( 11.3)	31 ( 15.1)	0.71 ( 0.28- 1.81)		0.628
Over dominant					
CC+TT	20 ( 37.8)	78 ( 38.0)	1		
CT	33 ( 62.2)	127 ( 62.0)	1.01 ( 0.54- 1.88)		1
Alleles					
C	61 ( 57.5)	221 ( 53.9)	1		
T	45 ( 42.5)	189 ( 46.1)	0.86 ( 0.56- 1.32)		0.573
rs920788 Polymorphism					
Codominant					
TT	37 ( 69.8)	156 ( 60.2)	1		
CT	14 ( 26.4)	96 ( 37.1)	0.61 ( 0.31-1.19)		0.201
CC	2 ( 3.8)	7 ( 2.7)	1.20 ( 0.24-6.03)		0.685
	Case	Control			
rs920788 Polymorphism					
Dominant					
TT	37 (69.8)	156 (60.2)	1		
CT+CC	16 (30.2)	103 (39.8)	0.65 (0.34- 1.23)	0.216	
Recessive					
TT+CT	51 (96.2)	252 (97.3)	1		
CC	2 (3.8)	7 (2.7)	1.41 (0.28-6.99)	0.652	
Over dominant					
TT+CC	39 (73.6)	163 (97.3)	1		
CT	14 (26.4)	96 (37.1)	0.60 (0.31- 1.80)	0.157	
Alleles					
T	88 (83)	408 (78.8)	1		
C	18 (17)	110 (21.2)	0.75 (0.43- 1.31)	0.357	

## Discussion

LncRNAs have a key role in the tumor progression such as cell proliferation, migration, invasion, and drug resistance (Rajagopal et al., 2020). It has been suggested that they can be function as oncogenes or tumor suppressors genes. HOTAIR is a trans-acting lncRNA could regulate gene expression through chromatin remodeling, genome packaging and genome rearrangement (Bayram et al., 2015). In the present study the association of HOTAIR polymorphisms and lymphoma risk in an Iranian population were evaluated. We found that rs1899663 significantly decreased the risk of NHL in codominant, dominant, overdominant and allelic inheritance models. Meanwhile HOTAIR rs12826786, rs920788 and rs4759314 variants showed no association with NHL risk. We didn’t find any association between HOTAIR polymorphisms and HL. These results are similar to the previous work of our team. In prior study we found that rs12826786 and rs1899663 polymorphisms decreased the risk of breast cancer but rs920778 polymorphism demonstrated significant positive association with breast cancer; and the rs4759314 variant was not associated with breast cancer risk (Hassanzarei et al., 2017). Contradictory to our finding kim et al showed that distribution of rs7958904 and rs1899663 polymorphisms were significantly different in patients with colorectal cancer (CRC) from healthy control group and increased the risk of CRC. In agreement with us they didn’t find any association between rs4759314 and rs920778 polymorphisms and CRC (Kim et al., 2020). In addition, they demonstrated that rs1899663 TT genotype was associated with increased colon cancer mortality (Kim et al., 2020). Tung et al found no significant differences in genotype distributions of HOTAIR polymorphisms between urothelial cell carcinoma (UCC) patients and controls but they observed that SNP rs4759314 was significantly associated with an increased UCC risk and poor overall survival in younger and female subjects (Tung et al., 2019). They concluded the rs4759314 polymorphism is located in intron 1 which can affect the activity of promoter and expression of HOTAIR (Tung et al., 2019). The pooled results of a meta-analysis showed the rs7958904 polymorphism of HOTAIR significantly decreased susceptibility to overall cancer risk among five genetic models, while the rs920778 and the rs12826786 polymorphisms increased susceptibility to cancer risk in all genetic models. Also, no significant association was found between the rs1899663, rs874945, and rs4759314 polymorphisms and susceptibility of cancer (Li et al., 2018). Liu et al in a meta-analysis demonstrated rs7958904 polymorphism decreased cancer risk but rs920778 polymorphism similarly rs4759314 and rs874945 polymorphisms, increased risk of cancer. Also No association was found between rs1899663 polymorphism and cancer susceptibility (Liu et al., 2017). Recently in a meta-analysis Moazeni-Roodi et al showed that rs4759314 polymorphism significantly increased the risk of gastric cancer but they did not find any association between this polymorphism and gastrointestinal (GI) cancer, breast cancer, pancreatic cancer and lung cancer. Furthermore they revealed rs920778 polymorphism significantly increased the risk of GI cancer and gastric cancer however no association was seen between this polymorphism and susceptibility to breast cancer. Eventually Subgroup analysis by cancer type indicate the rs1899663 and rs7958904 polymorphisms was not association with cancer susceptibility (Moazeni-Roodi et al., 2020). According to these results we propose that the effect of these polymorphisms is dependent on different cancer types. Yan et al., (2016)confirmed the high expression of HOTAIR in lymphoma tissues promotes cell proliferation and associated with poor prognosis and lower survival of patients with B cell lymphoma. Lin et al., (2018) in a meta- analysis showed elevated expression of HOTAIR in patients with leukemia and lymphoma accompanied with poor overall survival. Indeed HOTAIR interacts with PRC2 eventually decrease expression of multiple genes that acts as tumor suppressor genes (Chiyomaru et al., 2014; Ke et al., 2015).

In conclusion, our finding suggests that polymorphism in HOTAIR rs1899663 plays a protective role in susceptibility to NHL in southeast of Iran. Because to the best of our knowledge this is the first research concerning the HOTAIR polymorphisms and the risk of lymphoma susceptibility in the literature more studies with larger sample size in patients of different ethnic origins should be done to confirm our findings. 

## Author Contribution Statement

MT, HAS conceived and designed the study; SK, BM and GHB performed the experiments and analyzed the data; SMH performed data collection. All authors contributed to the writing of the manuscript and reviewed and approved the final manuscript.

## Conflict of interest

All authors declare no conflict of interest.
